# Vascular, but not luminal, activation of FFAR1 (GPR40) stimulates GLP-1 secretion from isolated perfused rat small intestine

**DOI:** 10.14814/phy2.12551

**Published:** 2015-09-17

**Authors:** Louise W Christensen, Rune E Kuhre, Charlotte Janus, Berit Svendsen, Jens J Holst

**Affiliations:** 1 Novo Nordisk Foundation Center for Basic Metabolic Research and Department of Biomedical Sciences, Panum Institute, University of CopenhagenCopenhagen, Denmark

**Keywords:** G-protein-coupled receptor, incretin, long-chain fatty acids

## Abstract

Glucagon-like peptide 1 (GLP-1) plays a central role in modern treatment of type 2 diabetes (T2DM) in the form of GLP-1 enhancers and GLP-1 mimetics. An alternative treatment strategy is to stimulate endogenous GLP-1 secretion from enteroendocrine L cells using a targeted approach. The G-protein-coupled receptor, FFAR1 (previously GPR40), expressed on L cells and activated by long-chain fatty acids (LCFAs) is a potential target. A link between FFAR1 activation and GLP-1 secretion has been demonstrated in cellular models and small-molecule FFAR1 agonists have been developed. In this study, we examined the effect of FFAR1 activation on GLP-1 secretion using isolated, perfused small intestines from rats, a physiologically relevant model allowing distinction between direct and indirect effects of FFAR1 activation. The endogenous FFAR1 ligand, linoleic acid (LA), and four synthetic FFAR1 agonists (TAK-875, AMG 837, AM-1638, and AM-5262) were administered through intraluminal and intra-arterial routes, respectively, and dynamic changes in GLP-1 secretion were evaluated. Vascular administration of 10 μmol/L TAK-875, 10 μmol/L AMG 837, 1 μmol/L and 0.1 μmol/L AM-1638, 1 μmol/L AM-6252, and 1 mmol/L LA, all significantly increased GLP-1 secretion compared to basal levels (*P *<* *0.05), whereas luminal administration of LA and FFAR1 agonists was ineffective. Thus, both natural and small-molecule agonists of the FFAR1 receptor appear to require absorption prior to stimulating GLP-1 secretion, indicating that therapies based on activation of nutrient sensing may be more complex than hitherto expected.

## Introduction

Glucagon-like peptide 1 (GLP-1) is a gut hormone that has gained a central position in modern treatment of type 2 diabetes mellitus (T2DM). Currently, the therapeutic effects of GLP-1 are achieved through injectable GLP-1 analogs (GLP-1 or incretin mimetics) and oral GLP-1 enhancers, that is, DPP-4 inhibitors inhibiting enzymatic degradation of endogenous GLP-1 (Drucker and Nauck 2006). As GLP-1 is secreted from enteroendocrine L cells embedded in intestinal mucosal epithelium (Eissele et al. 1992; Orskov et al. 1996), an alternative treatment strategy is to stimulate endogenous GLP-1 release through oral L-cell secretagogues. Increased postprandial levels of endogenous GLP-1 following the bariatric procedure, Roux-en-Y gastric bypass (RYGB) (Korner et al. 2007), have already been linked to the substantial weight loss and the remission of type 2 diabetes observed postsurgery (le Roux et al. 2006; Jorgensen et al. 2012). This indicates that there is an extra capacity in the GLP-1 axis which could be mobilized through pharmacological L-cell stimulation. Physiologically, GLP-1 is secreted in response to ingestion of nutrients with dietary fat constituting a potent L-cell stimulus (Elliott et al. 1993; Beglinger et al. 2010). This secretory response may be, at least partly, mediated through activation of free fatty acid receptor 1 (FFAR1, previously GPR40) – a G-protein-coupled receptor expressed on L cells (Edfalk et al. 2008; Xiong et al. 2013) recognizing long-chain free fatty acids (LCFA) released from hydrolyzed dietary fat (Briscoe et al. 2003; Kotarsky et al. 2003; Itoh and Hinuma 2005). FFAR1 constitutes an attractive antidiabetic drug target as this receptor is also expressed on pancreatic *β*-cells (Briscoe et al. 2003; Tomita et al. 2006; Del Guerra et al. 2010; Yashiro et al. 2012) in which FFAR1 activation potentiates glucose-stimulated insulin secretion (GSIS) (Itoh et al. 2003; Latour et al. 2007; Schnell et al. 2007; Kebede et al. 2008; Alquier et al. 2009). A number of FFAR1 selective agonists have been developed (Negoro et al. 2010; Lin et al. 2011; Houze et al. 2012; Wang et al. 2013) and several studies have confirmed the antidiabetic potential of FFAR1 activation through the direct insulinotropic effect on the *β*-cells (Christiansen et al. 2008; Tan et al. 2008; Lin et al. 2011; Tsujihata et al. 2011; Araki et al. 2012; Burant et al. 2012; Yashiro et al. 2012; Kaku et al. 2013).

A stimulatory effect of FFAR1 activation on GLP-1 secretion has also been reported in single cell studies and in some rodent studies (Reimann et al. 2008; Parker et al. 2009; Luo et al. 2012; Habib et al. 2013; Xiong et al. 2013), while clinical studies show inconclusive results regarding the effect of FFAR1 agonists on GLP-1 secretion (Leifke et al. 2012; Kaku et al. 2013). A detailed investigation of the direct link between FFAR1 activation and GLP-1 secretion in a physiologically relevant model has not been carried out. We, therefore, explored the effect of FFAR1 activation on GLP-1 secretion in rats using the isolated, in situ perfused small intestine. Unlike cell cultures (primary as well as cell lines) in which cells have lost their polarity, natural intercell association, and neurovascular supply, the endocrine cells in the perfused model are studied in their natural environment, allowing physiologically relevant studies of secretory mechanisms. In the present investigation, we studied dynamic changes in GLP-1 secretion in response to administration of the endogenous FFAR1 ligand, linoleic acid (LA), as well as synthetic selective FFAR1 agonists. Stimulants were administered from either the luminal or vascular side of the perfused intestine.

## Methods

### Ethical approval

This study was carried out with permission from the Danish Animal Experiments Inspectorate (*license no.:* 2013-15-2934-00833) and the local ethical committee (EMED, P-13-240) in accordance with the guidelines of Danish legislation governing animal experimentation.

### Animals and perfusion protocol

Male Wistar rats aged 8–10 weeks were purchased from Taconic (Ejby, Denmark) and housed in pairs under a 12 h light–dark cycle with ad libitum access to water and standard chow. Nonfasted rats weighing 250 g (CV 7%) were anesthetized by subcutaneous injection with Hypnorm®/Midazolam (0.079 mg fentanyl citrate + 2.5 mg fluanisone + 1.25 mg midazolam) and placed on a heating table at 37°C. The abdominal cavity was opened with two oblique incisions and colon plus 2/3 of the small intestine were resected leaving 32 cm (CV 19%) of the proximal small intestine in situ. The vascular supply to the duodenum and pancreas was ligated and a plastic tube was inserted proximally into the gut lumen allowing a gentle flush with 37°C perfusion buffer (PB) to remove intestinal contents. A constant luminal flow of 37°C PB (0.150 mL/min) was maintained. Using a universal single-pass perfusion system (UNIPER UP-100, Hugo Sachs Elektronik-Harvard apparatus, March-Hugstetten, Germany), the gut was vascularly perfused (7.5 mL/min) via a cannula inserted into in the superior mesenteric artery. Venous effluent was collected via the cannulated portal vein. The rat was sacrificed by exsanguination immediately after vascular perfusion was established. The perfused intestine was allowed to equilibrate for 30 min before experimental protocols were initiated. Venous effluents were collected each minute and samples were immediately put on ice and stored at –20°C until analysis. As a live assessment of gut health, perfusion pressure was recorded continuously by a pressure transducer (APT300, Hugo Sachs Elektronik) and random samples of PB from both the arterial and venous side pre- and postprotocol were analyzed for respiratory parameters (Po_2_, Pco_2_, pH, and lactate) using a blood gas analyzer (ABL700, Radiometer, Copenhagen, Denmark).

In randomly selected perfusion experiments (*n *=* *17), an approximately 1-cm midsegment of the perfused intestine was excised after termination of the experiment and stored in formaldehyde (4°C) for later histological examination (paraffin embedded and hematoxylin/eosin stained as described previously) (Kissow et al. 2012).

### Stimulations

Luminal stimuli were administered as a 5 mL bolus (2.5 mL/min) followed by continuous infusion (0.150 mL/min) throughout the remaining stimulation period. To terminate luminal stimulation, a 5 mL flush of PB (2.5 mL/min) was administered to clear the gut lumen of stimulatory solutions. Vascular stimuli were administered intra-arterially. All experiments were completed by vascular administration of 10 nmol/L bombesin (BBS). There were no negative responders to BBS. Each perfusion experiment was executed in a fresh preparation applying either vascular or luminal stimulatory routes, respectively.

### Hormone measurement

GLP-1 concentration in venous effluents was determined by in-house radioimmunoassay (RIA) employing a rabbit antiserum directed against the amidated C-terminus of GLP-1 (code no. 89390), thus detecting the active isoform, GLP-1_7-36amide_, and its metabolite, GLP-1_9-36amide_, as described previously (Orskov et al. 1994). Applied dilution was ×30,000. Assay detection limit was 1 pmol/L. As the intestine was perfused at a constant rate, GLP-1 concentrations parallel GLP-1 output. Recent studies showed that GLP-1 is predominantly amidated in rodents (Kuhre et al. 2014a; Svendsen et al. 2014). Therefore, measures of amidated GLP-1 isoforms were interpreted as total GLP-1 in this study, although a small contribution by GLP-1 7(9)-37 would be missed.

### Reagents

Reagents were purchased from Sigma-Aldrich (Broendby, Denmark) unless otherwise stated. PB consisted of a modified Krebs–Ringer bicarbonate buffer containing 0.1% human serum albumin (HSA) from Millipore (Billerica, MA), 5% dextran T-70 from Pharmacosmos (Holbaek, Denmark), 3.5 mmol/L glucose, and 5 mmol/L pyruvate, fumarate, and glutamate, respectively. Prior to every experiment, PB used in the vascular perfusion circuit was added to a mixture of amino acids, Vamin® (0.5 mL/100 mL) and 3-isobutyl-1-methylxanthine (IBMX) to a final concentration of 10 μmol/L.

IBMX (a phosphodiesterase inhibitor) was applied to sensitize the secretory mechanisms in the perfused gut. PB was oxygenated with a gas mixture of 95% O_2_ and 5% CO_2_. FFAR1 agonists were a gift from Merck (Kenilworth, NJ) and included partial agonists, TAK-875 (Negoro et al. 2010) and AMG 837 (Hauge et al. 2014) and full agonists, AM-1638 (Brown et al. 2011, 2012; Lin et al. 2012) and AM-5262 (Wang et al. 2013). FFAR1 agonists and IBMX were dissolved in dimethyl sulfoxide (DMSO) and further diluted in PB before experimental use. The perfused intestine was not exposed to DMSO concentrations above 1% (which did not significantly stimulate hormone secretion in control experiments, Fig. 3E and F, *n* = 6). BBS, which is a well-known GLP-1 secretagogue and thus served as a positive control, was dissolved in distilled water containing 1% HSA. The long-chain free fatty acid, LA, was administered in different formulations for vascular and luminal stimulations, respectively. For vascular stimulation, two LA formulations were tested: LA solubilized in 100% DMSO, which prior to experimental use was diluted in PB into a final LA concentration of 1 mmol/L (slightly opalescent solution), and “LA water soluble” (cat. no. L5900), that is, LA solubilized with methyl-*β*-cyclodextrin (MBCD), which was further diluted in PB into a final LA concentration of 100 μmol/L (clear formulation). LA for luminal stimulation was administered as sodium linoleate, “LA water soluble” or as mixed lipid micelles, respectively. Sodium linoleate powder was dissolved in isotonic saline (0.9%) into a final LA concentration of 10 mmol/L (clear formulation) by gently stirring at room temperature for 1 h and finally securing a final pH ≈ 9.5 as described previously for sodium oleate (Beglinger et al. 2010). “LA water soluble” for luminal stimulation was dissolved in isotonic saline into a final LA concentration of 1 mmol/L (clear formulation). The lipid micelle formula was based on a previous study (Chateau et al. 2005). For preparation of 1 mL lipid micelles, 0.6 *μ*L of 100 mmol/L oleic acid, 0.2 *μ*L of each 100 mmol/L l-*α*-lysophosphatidylcholine, 100 mmol/L 2-mono-oleoylglycerol, and 25 mmol/L cholesterol were mixed in a sterile glass tube, dried under stream of nitrogen, and dissolved in 83 *μ*L saline containing 0.1% fatty acid-free bovine serum albumin (BSA) and 24 mmol/L sodium taurocholate. Saline containing 0.1% fatty acid-free BSA was added up to 1 mL.

### Statistics

Statistical evaluation was performed using GraphPad Prism 5 software (La Jolla, CA). Data are presented as mean values ±1 SEM. Statistical significance was assessed by paired *t*-test, comparing average hormone concentration of the initial 10-min baseline period with the initial 10 min of stimulation periods. Respective sample sizes are presented in the Results section. For intergroup comparisons of vascular stimulations with LA, TAK-875, and AMG 837, respectively, to vehicle (DMSO 1%) unpaired *t*-tests were performed. *P*-values <0.05 were considered statistically significant.

## Results

### Vascular, but not luminal, administration of linoleic acid stimulates GLP-1 release from the perfused rat small intestine

**Figure 1:**
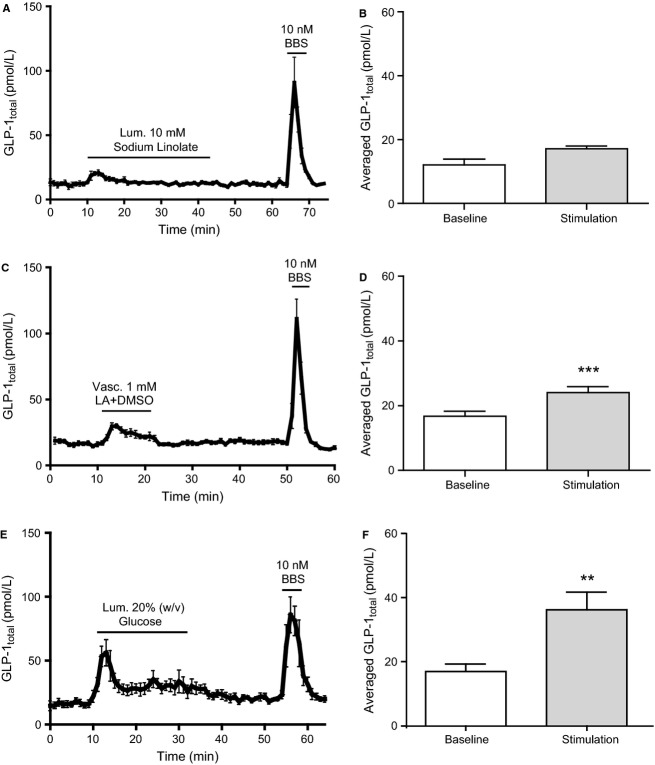
Vascular, but not luminal, linoleic acid stimulates GLP-1 secretion from perfused rat small intestine. Data are shown as mean values ±1 SEM. Dynamic changes in GLP-1_total_ concentrations are depicted in response to (A) luminal (10 mmol/L) sodium linoleate, (C) vascular (1 mmol/L) linoleic acid (DMSO 1%), and (E) luminal 20% w/v glucose (pos. luminal control) and as average GLP-1_total_ concentrations at baseline or in response to (B) luminal (10 mmol/L) sodium linoleate, (D) vascular (1 mmol/L) linoleic acid (DMSO 1%), and (F) luminal 20% w/v glucose. Bombesin (BBS) were included as positive control. Statistical significance between stimulant and baseline levels was assessed by paired *t*-test. **P <* *0.01, ***P <* *0.001, *n *=* *6 (A, B), *n *=* *7 (C, D), *n *=* *8 (E, F).

**Figure 2:**
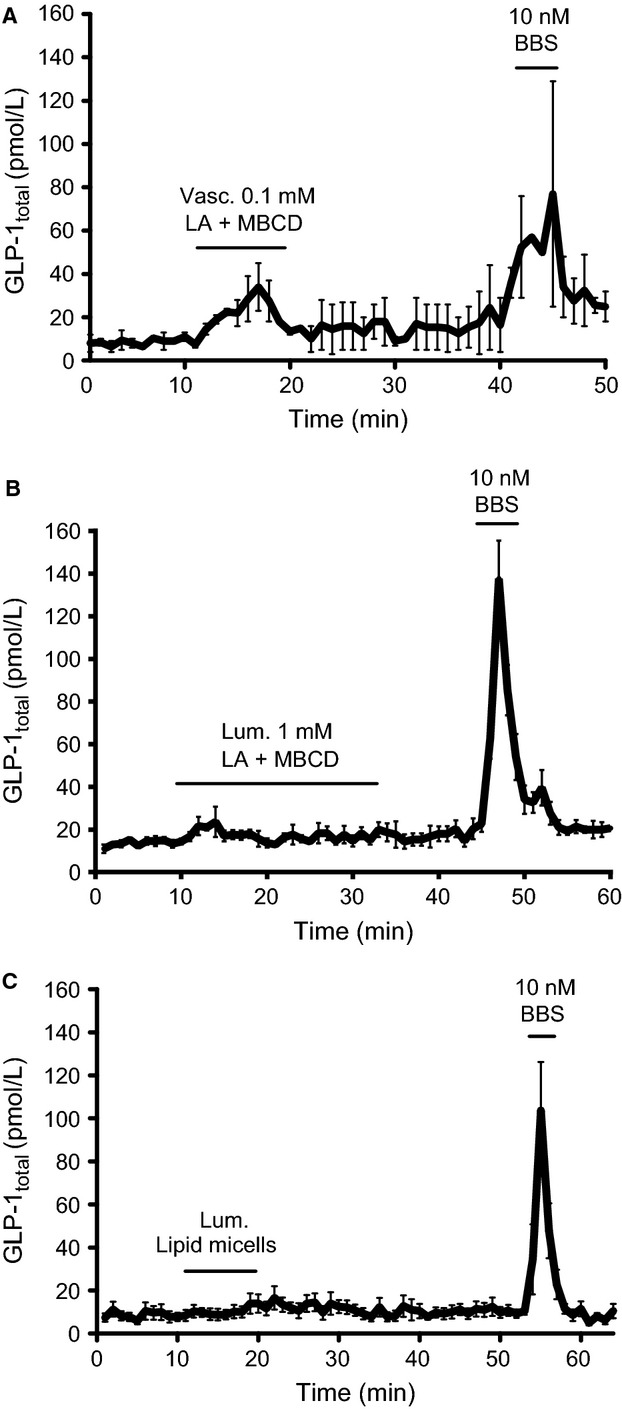
Luminal administered lipid micelles and methyl-*β*-cyclodextrin (MBCD) solubilized linoleic acid do not stimulate GLP-1 secretion, whereas vascular administered methyl-*β*-cyclodextrin solubilized linoleic acid increased GLP-1 secretion. Data are shown as mean values ±1 SEM. Dynamic changes in GLP-1 total concentrations are depicted in response to (A) vascular stimulation with 0.1 mmol/L and (B) luminal stimulation with 1 mmol/L linoleic acid solubilized with MBCD and (C) mixed lipid micelles. Statistical significance was not assessed for these data, *n *=* *2 (A), *n *=* *3 (B), *n *=* *5 (C).

Vascular administration of 1 mmol/L LA + DMSO significantly increased venous GLP-1 concentration compared to baseline (from 16.7 ± 4.0 pmol/L to 24.0 ± 4.8 pmol/L, *P *<* *0.001, *n *=* *7) (Fig. 1C and D). In contrast, luminal administration of 10 mmol/L sodium linoleate minimally affected GLP-1 concentrations (from 12.1 ± 1.8 pmol/L to 17.1 ± 1.0 pmol/L, *P *=* *0.07, *n *=* *6) (Fig. 1A and B). Consistently, luminal administration of LA + MBCD and mixed lipid micelles, respectively, did not appear to affect GLP-1 secretion, while vascular LA + MBCD elicited a marked GLP-1 response (Fig. 2A–C). As a positive luminal control, 20% w/v luminal glucose caused a significant increase in GLP-1 concentration (from 16.9 ± 2.4 pmol/L to 36.2 ± 5.6 pmol/L, *P *<* *0.01, *n *=* *8) (Fig. 1E and F). In all experiments, BBS was administered intravascularly as a positive control. BBS elicited robust GLP-1 responses in all experiments (Figs. 5).

**Figure 3:**
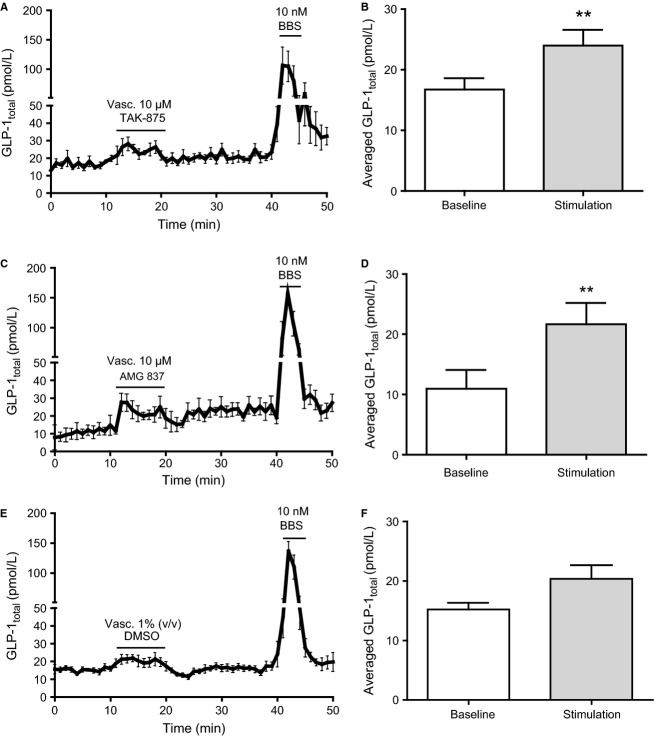
Vascular administration of partial GPR40 agonists stimulates GLP-1 secretion from perfused rat small intestine. Data are shown as mean values ±1 SEM. Dynamic changes in GLP-1_total_ concentrations are depicted in response to vascular stimulation with (A) 10 μmol/L TAK-875, (C) 10 μmol/L AMG 837, and (E) 1% DMSO (vehicle) and as average GLP-1_total_ concentrations at baseline or in response to vascular stimulation with (B) 10 μmol/L TAK-875, (D) 10 μmol/L AMG 837, and (F) vehicle. Statistical significance between stimulant and baseline levels was assessed by paired *t*-test. **P <* *0.01, *n *=* *6.

**Figure 4:**
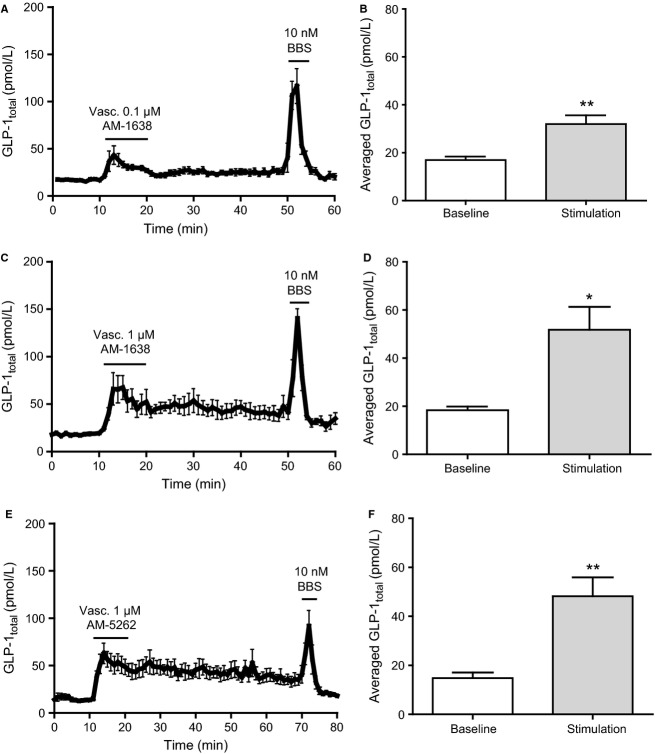
Vascular administered full GPR40 agonists stimulate GLP-1 secretions from isolated perfused rat small intestine. Data are shown as mean values ±1 SEM. Dynamic changes in GLP-1_total_ concentrations are depicted in response to vascular stimulation with (A) 0.1 μmol/L AM-1638, (C) 1 μmol/L AM-1638, and (E) 1 μmol/L AM-5262 and as average GLP-1_total_ concentrations at baseline or in response to vascular stimulation with (B) 0.1 μmol/L vascular AM-1638, (D) 1 μmol/L vascular AM-1638, and (F) 1 μmol/L vascular AM-5262. Statistical significance between stimulant and baseline levels was assessed by paired *t*-test. *P <* *0.05, **P <* *0.01, *n *=* *6 (A, B), *n *=* *7 (C–F).

**Figure 5:**
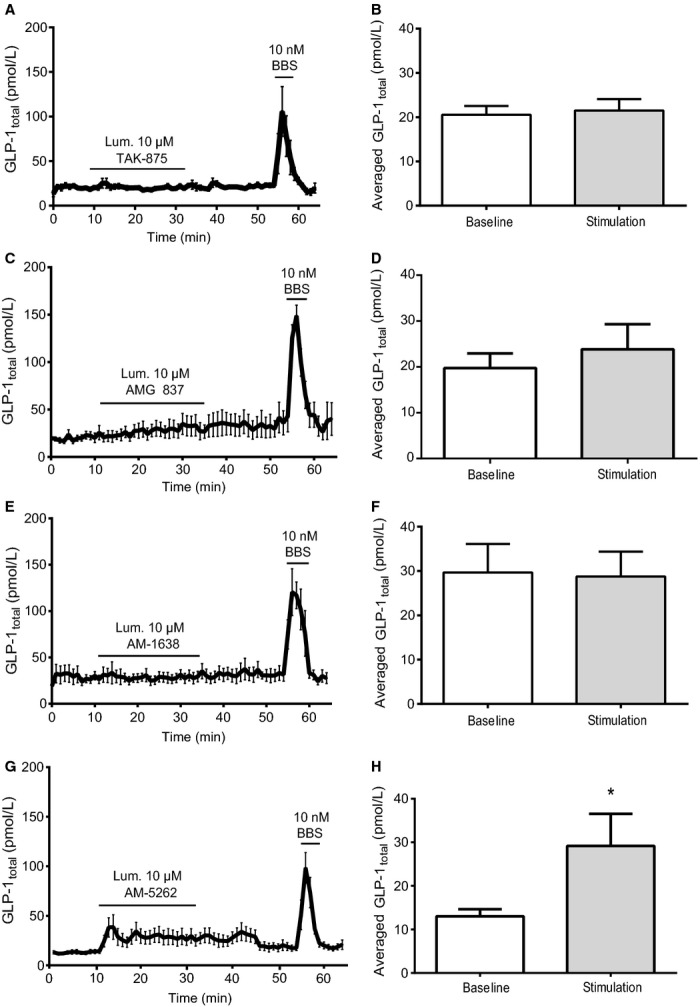
Luminal administration of GPR40 agonists does not stimulate GLP-1 secretion from perfused rat small intestine. Data are shown as mean values ±1 SEM. Dynamic changes in GLP-1_total_ concentrations are depicted in response to luminal stimulation with (A) 10 μmol/L TAK-875, (C) 10 μmol/L AMG 837, (E) 10 μmol/L AM-1638 and (G) 10 μM AM-5262 and as average GLP-1_total_ concentrations at baseline or in response to luminal stimulation with (B) 10 μmol/L TAK-875, (D) 10 μmol/L AM-837, (F) 10 μmol/L AM-1638 and (H) 10 μM AM-5262. Statistical significance between stimulant and baseline levels was assessed by paired *t*-test. *P <* *0.05, *n* = 6 (A-F), *n* = 7 (G,H).

### Vascular, but not luminal, selective FFAR1 activation stimulates GLP-1 release from the perfused rat small intestine

Partial FFAR1 agonists administered intravascularly at 10 μmol/L (final concentration) induced a modest but significant increase in GLP-1 concentration (TAK-875: from 16.7 ± 1.8 pmol/L to 24.0 ± 2.6 pmol/L, AMG 837: from 11.0 ± 3.1 pmol/L to 21.7 ± 3.5 pmol/L, both *P *<* *0.01 compared to respective baselines, *n *=* *6) (Fig. 3A–D). In control experiments, vascular administration of vehicle (DMSO 1%) weakly and insignificantly increased GLP-1 concentration (from 15.2 ± 1.1 pmol/L to 20.4 ± 2.3 pmol/L, *P *=* *0.08, *n *=* *6) (Fig. 3E and F). Intergroup comparisons between LA, TAK-875, AMG 837, and DMSO stimulations showed that only the GLP-1 response induced by vascular AMG 837 was significantly different from the vehicle control (*P *<* *0.05). Intravascular administration of full FFAR1 agonists elicited a robust GLP-1 response (AM-1638 [0.1 μmol/L]: from 16.9 ± 1.4 pmol/L to 32.0 ± 3.7 pmol/L, *P *<* *0.01, *n *=* *6; AM-1638 [1 μmol/L]: from 18.4 ± 1.5 pmol/L to 51.8 ± 9.5 pmol/L, *P *<* *0.05, *n *=* *7; and AM-5262 [1 μmol/L]: 14.8 ± 2.2 pmol/L to 48.2 ± 7.7 pmol/L, *P *<* *0.01, *n *=* *8) (Fig. 4A–F).

Surprisingly, administration of AM-1638 and AM-5262 at 1 μmol/L final concentration both continuously stimulated GLP-1 secretion long after the infusion periods ended. Luminal administration of TAK-875, AMG 837, and AM-1638 (all at 10 μmol/L), respectively, did not affect GLP-1 secretion (TAK-875: from 20.5 ± 2.0 pmol/L to 21.5 ± 2.6 pmol/L, AMG 837: from 19.7 ± 3.2 pmol/L to 23.8 ± 5.5 pmol/L, and AM-1638: from 29.7 ± 6.4 pmol/L to 28.8 ± 5.6 pmol/L, all *P *>* *0.05, *n *=* *6) (Fig. 5). For luminal AM-5262 stimulation, a 10-fold higher concentration (10 μmol/L) compared to the vascular stimuli, produced a small, but significant, prolonged response (from 13.01 ± 1.6 to 29.16 ± 7.8, *P* = 0.03, *n* = 7), (Fig. 5G, H). This was interpreted as a response to absorption of the agonist from the luminal and into the interstitial/vascular compartment.

## Discussion

The main finding of this study is that vascular FFAR1 activation stimulates GLP-1 secretion, whereas luminal FFAR1 stimulation is without effect in the isolated perfused rat small intestine.

In view of the therapeutic success of current GLP-1 based strategies in treatment of T2DM, the prospect to harness endogenous GLP-1 production from enteroendocrine L cells using a targeted approach has emerged (Albrechtsen et al. 2014). To do so, the secretory mechanisms underlying meal-induced GLP-1 release must be identified. Fat is a known stimulator of GLP-1 secretion (Elliott et al. 1993; Beglinger et al. 2010; Lindgren et al. 2011). Orlistat, an inhibitor of gastric and pancreatic lipase, has been demonstrated to attenuate postprandial GLP-1 release in humans, suggesting that hydrolysis of triglycerides into FFAs is an essential step in fat-induced GLP-1 secretion (Ellrichmann et al. 2008). Accordingly, luminal administered LCFA have been shown to stimulate GLP-1 secretion in humans (Feltrin et al. 2004; Carr et al. 2008; Beglinger et al. 2010) and rodents (Adachi et al. 2006; Dailey et al. 2010). This effect has been proposed to be mediated through activation of G-protein-coupled receptors as FFAR1 (Edfalk et al. 2008; Reimann et al. 2008; Parker et al. 2009; Xiong et al. 2013) and GPR120(Hirasawa et al. 2005). Selective FFAR1 activation seems to be a particular attractive target for the treatment of T2DM as the receptor not only colocalizes with the incretin hormones, GLP-1, and glucose-dependent insulinotropic polypeptide (GIP) (Edfalk et al. 2008; Liou et al. 2011), but appears to be present on the pancreatic *β*-cells as well. Thus, selective FFAR1 activation should theoretically give rise to a robust glucose-stimulated insulin secretion (GSIS) as the *β*-cells would be both directly and indirectly (incretins) activated. The direct link between FFAR1 activation and GLP-1 secretion has been investigated using the GLP-1 secreting cell line GLUTag and primary L-cell cultures (Luo et al. 2012; Habib et al. 2013). It is, however, an often neglected problem that these cells lose their natural polarity during the process of cultivation. In many tissues, polarity and contact to neighboring cells are essential for normal function. Consequently, the full functional responsiveness and secretory capacity of GLP-1-producing cells may not be preserved in these cellular models.

FFAR1 activation has also been linked to GLP-1 secretion in vivo in wild-type and/or FFAR1 deficient animals (Edfalk et al. 2008; Luo et al. 2012; Xiong et al. 2013). Although in intact animals it can be difficult to distinguish direct from indirect effects, and studies of hormone secretion are hampered by the limited volume of plasma that can be harvested, particularly from mice, often in combination with lack of suitable assays. In the case of FFAR1, a tissue-specific knock out model has not yet been introduced, and since the receptor is expressed in various tissues, including the central nervous system (Briscoe et al. 2003; Nakamoto et al. 2012), pancreatic *α*-cells (Flodgren et al. 2007), taste buds (Cartoni et al. 2010), and other enteroendocrine cells (GIP and cholecystokinin [CCK]) (Edfalk et al. 2008), the isolated effect of receptor activation or deletion in the intestine is difficult to evaluate in vivo. The isolated, perfused small intestine allows us to study intestinal dynamics in a physiological setting where the enteroendocrine cells maintain natural polarity and integration in an intact intestinal mucosa. In addition, the intestine is isolated from the systemic circulation which provides the means to investigate organ-specific responses without influences from whole body regulatory mechanisms. Isolated perfused small intestines have, to the best of our knowledge, not yet been used to approach the involvement of FFAR1 activation in GLP-1 secretion.

Using this particular method, we hereby show that vascular, but not luminal, FFAR1 activation significantly stimulates GLP-1 secretion. This was evident using the endogenous ligand, LA, as well as the synthetic agonists.

Intra-arterial infusion of LA significantly stimulated GLP-1 release compared to basal levels (Fig. 1C and D), while sodium linoleate administered luminally only elicited a transient nonsignificant peak in GLP-1 secretion (Fig. 1A and B). An intergroup comparison between vascular LA and the DMSO control suggested that the observed effect of vascular LA may have been attributed to DMSO. However, an evident GLP-1 response was also observed upon vascular, but not luminal, stimulation with LA + MBCD (Fig. 2), which strongly supports a vascular LA-induced FFAR1 activation. The absence of response to intraluminal LCFA is consistent with a previous study on isolated perfused rat small intestine, where 100 mmol/L of luminal oleate did not increase GLP-1 secretion (Dumoulin et al. 1998).

The finding that vascular and not luminal LA stimulates GLP-1 secretion contrasts with previous results from an in vivo rodent study, demonstrating increased GLP-1 plasma levels after intrajejunal infusion of LA (Dailey et al. 2010). However, in this particular study, GLP-1 plasma levels were measured as active hormone in trunk blood from sacrificed animals after a 5-day continuous intestinal LA infusion. As the active isoforms of GLP-1 have an ultrashort half-life (less than 2 min in humans and less in rodents) and nutrient-induced GLP-1 secretion should be measured postprandially rather than after chronic stimulation, these findings cannot be considered physiologically relevant. Clinical studies have shown increased GLP-1 secretion in response to oleate in some (Beglinger et al. 2010), but not in all studies (Hansen et al. 2011). In addition, GLP-1 secretion after oleate infusion may result from activation of neuroendocrine loops involving vagal nerve activity and secretion of CCK and GIP, which both have been shown to stimulate GLP-1 secretion (Fieseler et al. 1995; Rocca and Brubaker 1999).

In the present study, increased GLP-1 secretion was also observed upon vascular FFAR1 activation using four synthetic selective FFAR1 agonists (Figs. 3 and 4). The partial FFAR1 agonists, TAK-875(Negoro et al. 2010) and AMG 837(Lin et al. 2011), both elicited a similar, modest, yet significant, GLP-1 response upon vascular administration compared to basal levels (Fig. 3A–D), while luminal administration did not affect GLP-1 secretion (Fig. 5). Intergroup comparison to the DMSO control showed that only the GLP-1 response induced by vascular AMG 837 was significantly different from vascular vehicle. This is consistent with very recent findings demonstrating a modest effect of AMG 837 and TAK-875 on GLP-1 secretion in vitro (Hauge et al. 2014), while only little or no effect was seen after oral gavage in mice in vivo (Luo et al. 2012; Hauge et al. 2014). Clinical studies investigating the antidiabetic potential of TAK-875 showed inconclusive effects on postprandial GLP-1 plasma levels (Leifke et al. 2012; Kaku et al. 2013). In the present study, the full FFAR1 agonists, AM-1638 (Brown et al. 2012) and AM-5262 (Wang et al. 2013), both caused a pronounced GLP-1 response, at least comparable to the response induced by intraluminal glucose (Fig. 4A–F). The efficacy of AM-1638 and AM-5262 (Luo et al. 2012; Wang et al. 2013) may be a consequence of a combined *G*_s_ and *G*_q_ signaling activated by these agonists rather than *G*_q_ signaling alone (Hauge et al. 2014).

The dynamic measurements in the present study, surprisingly, revealed that GLP-1 secretion did not return to baseline after termination of infusion with 1 *μ*mol/L AM-1638 and AM-5262, respectively (Fig. 4C–F). At 0.1 μmol/L AM-1638, a defined GLP-1 response within the infusion period was observed (Fig. 4A and B) which may merely suggest that high agonist concentrations require long wash out periods. Luminal administration of AM-1638 did not affect GLP-1 secretion (Fig. 5E and F), but as mentioned above luminal stimulation with AM-5262 at a 10-fold higher concentration (10 μmol/L) than that causing a dramatic vascular stimulation produced a small, but significant, prolonged response (Fig. 5G, H). We interpreted this as a response to absorption of the agonist from the luminal and into the interstitial/vascular compartment.

In order to verify that the absence of response to luminal FFAR1 stimulation was not due to mucosal damage in luminally stimulated gut segments, mucosal integrity was confirmed by subsequent histological examinations of the perfused intestinal segments (data not shown). Furthermore, as a positive luminal control, we administered a 20% (w/v) luminal glucose solution, which is considered a robust stimulus of GLP-1 secretion and has previously been shown to induce a strong GLP-1 response from perfused rat small intestines (Dumoulin et al. 1998; Kuhre et al. 2014b). Consistent with these studies, intraluminal glucose infusion caused a pronounced GLP-1 response (Fig. 1E and F). Moreover, luminal administration of protein hydrolysates has recently been shown to stimulate GLP-1 secretion in the same preparation (Svendsen et al. 2014). However, LCFA may interact differently from glucose and protein hydrolysates with the luminal intestinal milieu due to their lipophilic nature. To exclude lack of solubility of LCFA as an explanation for the lack of GLP-1 secretion upon luminal FFAR1 activation, different solubilization methods were tested in pilot experiments. In addition to the administration of luminal lineolate (Fig. 1A), we explored luminal stimulation with LA solubilized with MBCD. Despite increasing GLP-1 secretion upon vascular administration (Fig. 2A), this formulation did not stimulate GLP-1 secretion when administered intraluminally (Fig. 2B). Finally, to mimic the complex physiological intestinal transport of LCFA, luminal stimulation with mixed lipid micelles was investigated. This luminal stimulus also did not increase GLP-1 secretion (Fig. 2C).

Based on these futile efforts to activate GLP-1 secretion upon intraluminal administration of LA solutions, we conclude that it is not a hindered interaction of lipophilic luminal stimulants with FFAR1 that underlies the lack of GLP-1 response to luminal FFAR1 activation. Rather our findings could point to a predominant expression of FFAR1 in the basolateral L-cell membrane facing the intercellular compartment and the blood stream. In this case, physiological LCFA-induced GLP-1 secretion may occur secondary to intestinal lipid absorption. Supporting this notion, intraluminal lipid stimulation along with pharmacological inhibition of enterocyte formation of chylomicrons (Lu et al. 2012) and lipoproteins (Hata et al. 2011) resulted in reduced lipid-induced GLP-1 secretion. Identification of the exact membrane localization of L-cell FFAR1 would require advanced histological examination which was beyond the scope of this study. Thus, it appears that L-cell receptor activation not only requires absorption of the ligands, but possibly also appearance in the blood stream.

In conclusion, in order to develop pharmacological strategies for L-cell stimulation, we need to clarify the secretory mechanisms underlying nutrient-induced GLP-1 secretion. The isolated perfused small intestine is a robust model to explore such mechanisms. Because of the preserved polarity of the perfused preparation, we were able to demonstrate that vascular rather than luminal activation of FFAR1 stimulated GLP-1 secretion. Although our findings are consistent with the notion that oral FFAR1 agonists may constitute future L-cell stimulants for T2DM and obesity treatment, they also show that the mechanisms involved are more complicated than anticipated and appears to require appearance of the FFAR1 ligands in the circulation.
